# The Effect of Hydrogen on Plastic Anisotropy of Mg and α-Ti/Zr from First-Principles Calculations

**DOI:** 10.3390/ma16083016

**Published:** 2023-04-11

**Authors:** Jiwei Wang, Bin Shao, Debin Shan, Bin Guo, Yingying Zong

**Affiliations:** National Key Laboratory for Precision Hot Processing of Metals, School of Materials Science and Engineering, Harbin Institute of Technology, Harbin 150001, China

**Keywords:** hcp metals, hydrogen, ideal shear strength, anisotropy, first-principles

## Abstract

Mg and α-Ti/Zr exhibit high plastic anisotropy. In this study, the ideal shear strength across the basal, prismatic, pyramidal I, and pyramidal II slip systems in Mg and α-Ti/Zr with and without hydrogen was computed. The findings indicate that hydrogen reduces the ideal shear strength of Mg across the basal and pyramidal II slip systems, as well as of α-Ti/Zr across all four systems. Moreover, the activation anisotropy of these slip systems was analyzed based on the dimensionless ideal shear strength. The results suggest that hydrogen increases the activation anisotropy of these slip systems in Mg, while decreasing it in α-Ti/Zr. Furthermore, the activation possibility of these slip systems in polycrystalline Mg and α-Ti/Zr subjected to uniaxial tension was analyzed by utilizing the ideal shear strength and Schmidt’s law. The results reveal that hydrogen increases the plastic anisotropy of Mg/α-Zr alloy while decreasing that of α-Ti alloy.

## 1. Introduction

The dislocation slip is a dominant mode of plastic deformation in metals [1]. In the case of hexagonal close-pack (HCP) metals, such as Mg and α-Ti/Zr, there are multiple slip systems, including the basal <**a**> ({0001} <11−20>), prismatic <**a**> ({10−10} <11−20>), pyramidal I <**a**> ({10−11} <11−20>), and pyramidal II <**c**+**a**> ({11−22} <11−23>) slip systems. However, due to the HCP lattice’s asymmetry, the critical resolved shear stresses (CRSS, τ^CRSS^) for these slip systems are highly anisotropic [2]. For example, in Mg and its alloy, the τ^CRSS^ for the non-basal (prismatic and/or pyramidal) slip system is approximately 102 times higher than that for the basal slip system, which is considered the easiest slip system [3]. This anisotropy can be detrimental to ductility and formability [4], as the plastic deformation behavior is highly dependent on the direction of applied stress. However, alloying elements can modify the plastic anisotropy of these materials by affecting the activation of the slip systems [5,6,7,8]. For instance, rare earth elements can improve the formability of Mg alloys by promoting the activation of non-basal slip systems [9,10,11,12,13,14]. Hydrogen is a widely used alloying element in metals, but its effects on the mechanical properties of metals are generally considered deleterious. The introduction of hydrogen can lead to hydrogen embrittlement [15,16,17], which reduces the ductility and toughness of the metal. Nonetheless, hydrogen atoms can also have a positive impact on the mechanical properties of metals through hydrogen-enhanced plasticity (HEP) [18]. HEP occurs when hydrogen occupies sites in the metal crystal structure and reduces the metal’s shear modulus [19,20]. This reduction can decrease the interaction energy between dislocations and obstacles, enhancing the motion of dislocation in the metal [21,22]. Consequently, the metal exhibits increased levels of plastic deformation and improved energy absorption before fracture, leading to enhanced ductility and formability under certain conditions [23]. Notwithstanding significant advancements in the field, the fundamental mechanisms governing the influence of hydrogen on the mechanical properties of metals remain inadequately comprehended. As such, the present research endeavors to explore the impact of hydrogen on plastic deformation by investigating its effect on the activation anisotropy of typical slip systems. The ideal shear strength (ISS) is a rational physical quantity for quantifying the CRSS required for the activation of slip systems without disturbances from crystal defects such as vacancies and grain boundaries [24]. In light of this, this study investigated the impact of hydrogen on the activation anisotropy of typical slip systems in Mg and α-Ti/Zr, as well as the plastic anisotropy of these alloys under uniaxial tension using ISS derived from the first-principles calculation.

## 2. Computational Methods

**Figure 1:**
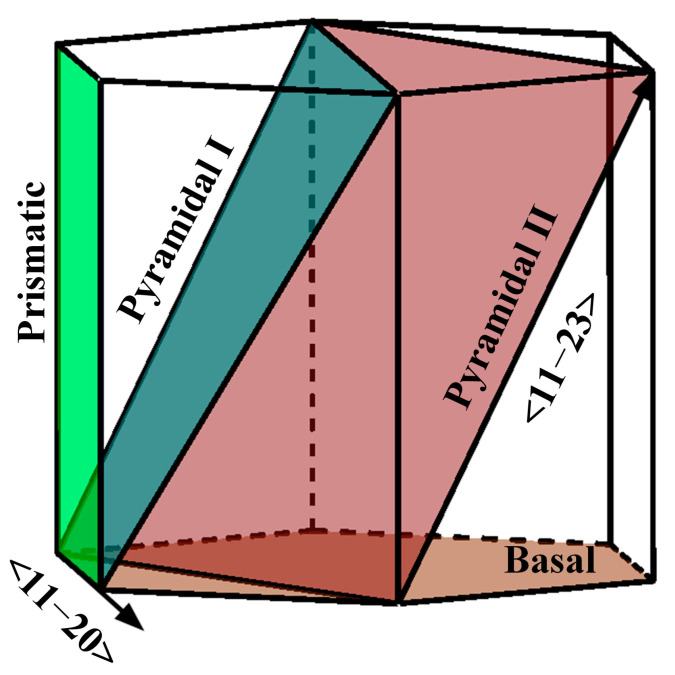
Schematic diagram of the applied shear strain direction for calculating the ISS.

The Kohn–Sham Density Functional Theory (KS-DFT), implemented in the Vienna Ab initio Simulation Package (VASP), was utilized to conduct the calculations [25,26]. The projector augmented-wave (PAW) method was utilized to describe electron–ion interactions [27]. Additionally, the Perdew–Burke–Ernzerhof (PBE) generalized that gradient functional [28] was employed to describe the exchange and correlation interactions. The plane-wave energy cutoff was thoughtfully chosen as 350 eV for Mg and 400 eV for α-Ti/Zr. In addition, the density of the Gamma-centered k-point mesh for Brillouin zone integration was set to 0.02. To determine the ISS, simple shear strains were applied along the slip direction on the slip plane in increments of 1% until yielding occurred. Additionally, the direction of the applied shear strain on each slip system is shown in Figure 1. At each step, the atomic positions and basis vectors were completely relaxed while the applied strain was kept fixed until the conjugate Hellmann–Feynman stresses of the other five independent strains reached a negligible level [29]. The relaxation process was performed using a quasi-Newton algorithm until the forces acting on all atoms were below 5 meV/Å and the total energy change was less than 10^−6^ eV. 4 × 2 × 2 (32 atoms) and 2 × 2 × 2 (16 atoms) periodicity were utilized for the supercell to achieve 3.125 and 6.25 at.% hydrogen content, respectively. The solid solution energy of hydrogen located at the tetrahedral interstitial site and octahedral interstitial site in bulk was calculated as follows: $E^{sol}=E_{bulk+H}-E_{bulk}-E_{H_{2}}$, where $E_{bulk+H}$ and $E_{bulk}$ are the total energy of supercell with and without hydrogen atom, respectively, $E_{H_{2}}$ is the total energy of hydrogen molecule in a vacuum. The preferred interstitial sites of hydrogen in Mg, α-Ti, and α-Zr were determined based on the minimum solution energy values obtained from the calculations, as presented in Table S1. The results indicated that hydrogen exhibited a preference for occupying the tetrahedral interstitial sites in Mg and α-Zr, whereas in α-Ti, hydrogen preferred the octahedral interstitial sites. These findings are consistent with previous research [19,30,31].

## 3. Results and Discussion

**Figure 2:**
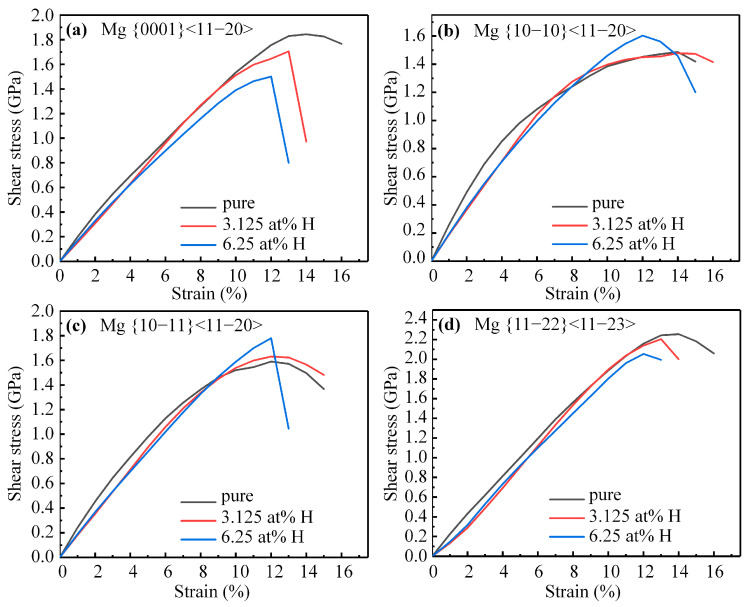
The stress–strain curves of Mg with or without hydrogen along (**a**) basal, (**b**) prismatic, (**c**) pyramidal I, and (**d**) pyramidal II slip systems.

The ISS of pure Mg across the basal, prismatic, pyramidal I, and pyramidal II slip systems were determined to be 1.84 GPa, 1.47 GPa, 1.60 Gpa, and 2.56 GPa, respectively. These results are consistent with previous research [24]. The ISS of Mg with hydrogen contents of 3.125 at. % and 6.25 at. % was calculated across these four slip systems, and the stress–strain curves are illustrated in Figure 2. The addition of hydrogen to Mg causes a remarkable decrease in the shear modulus (the slope of the stress–strain curve at zero strain) for all slip directions compared to pure Mg, with no further decrease observed with the higher hydrogen content. The impact of hydrogen on the ISS of Mg varies with the slip systems. Increasing the hydrogen content can systematically decrease the ISS across the basal and pyramidal II slip systems, but increase it across the prismatic and pyramidal I slip systems. Specifically, as illustrated in Figure 2a,d, the ISS across the basal and pyramidal II slip systems of Mg with 6.25 at. %H are reduced by 18.61% and 6.70%, respectively, relative to pure Mg. In contrast, as shown in Figure 2b,c, the ISS across the prismatic and pyramidal I slip systems of Mg with 6.25 at. %H increases by 7.81% and 11.97%, respectively, relative to pure Mg. The slip system with the minimum ISS among the four slip systems changes from prismatic to basal when the hydrogen content is 6.25 at. %. The presence of hydrogen also decreases the corresponding strain for the ISS across these slip systems, indicating that hydrogen weakens the shearability of a Mg alloy.

**Figure 3:**
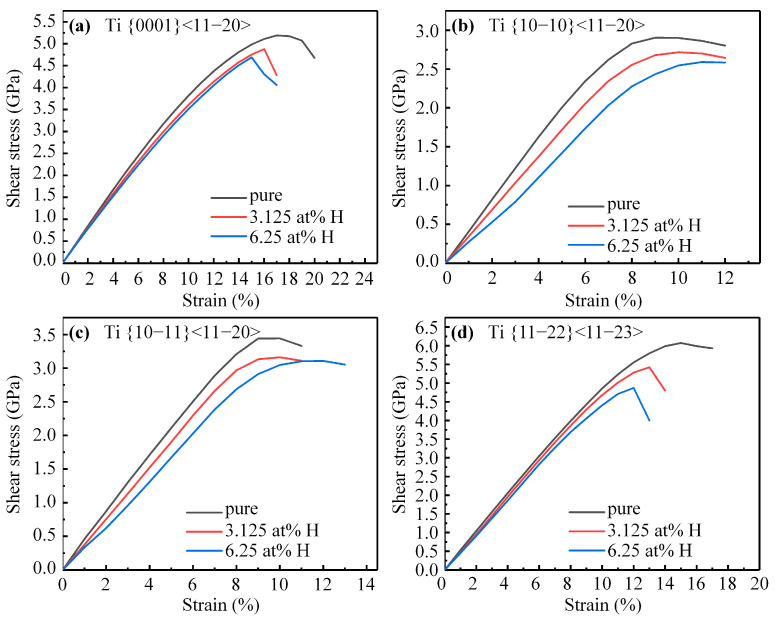
The stress–strain curves of α-Ti with or without hydrogen along (**a**) basal, (**b**) prismatic, (**c**) pyramidal I, (**d**) pyramidal II slip systems.

The ISS of pure α-Ti across four slip systems was determined to be 5.19 Gpa, 2.90 Gpa, 3.44 Gpa, and 6.07 GPa, respectively, which is in good agreement with the previous study [32]. The stress–strain curves of α-Ti with 3.125 at. %H and 6.25 at. %H are displayed in Figure 3. The addition of hydrogen led to a significant reduction in the shear modulus along all slip directions compared to pure α-Ti, and the ISS across all slip systems decreased with an increasing hydrogen content. Specifically, the ISS of α-Ti with 6.25 at. %H across those four slip systems decreased by 9.63%, 10.81%, 9.70%, and 19.77%, respectively, relative to pure α-Ti. The increasing hydrogen content has not altered the order of the ISS across those slip systems, and the ISS across the prismatic slip system remains at the minimum. The corresponding strains for the ISS across the basal and pyramidal II slip systems decrease with the increasing hydrogen content, while those for the ISS across the prismatic and pyramidal I slip systems increase. Therefore, the presence of hydrogen would increase the shearability of α-Ti on the prismatic and pyramidal I slip systems.

**Figure 4:**
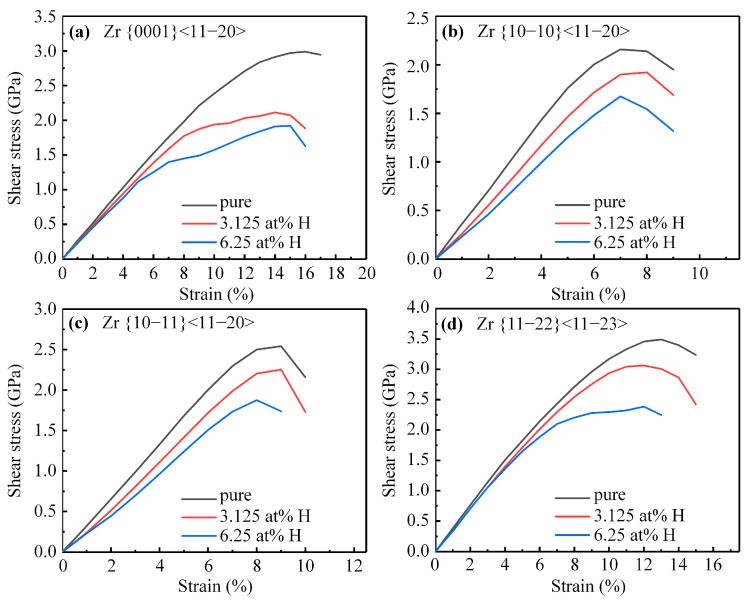
The stress–strain curves of α-Zr with or without hydrogen along (**a**) basal, (**b**) prismatic, (**c**) pyramidal I, (**d**) pyramidal II slip systems.

In the case of pure α-Zr, the ISS across the basal, prismatic, pyramidal I, and pyramidal II slip system is 2.99 GPa, 2.16 GPa, 2.54 Gpa, and 3.49 GPa, respectively. Similarly to the effect of hydrogen on α-Ti, the shear modulus and ISS of α-Zr across those four slip systems systematically decrease with an increasing hydrogen content, as shown in Figure 4. For instance, the ISS of α-Zr with 6.25 at. %H across those four slip systems reduced by 35.71%, 22.40%, 26.21%, and 31.67%, respectively, relative to pure α-Zr. Notably, the effect of hydrogen on reducing the ISS of α-Zr is more significant compared to that of α-Ti with 6.25 at. %H. Furthermore, the prismatic slip system consistently displays minimal ISS for α-Zr with the increasing hydrogen content.

**Figure 5:**
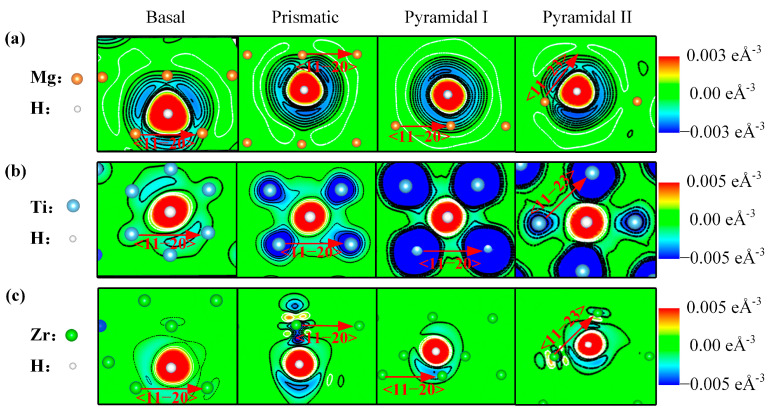
Plots of charge density difference on the basal, prismatic, pyramidal I, and pyramidal II slip systems around the hydrogen atom (white sphere) at the corresponding maximum shear stress in the (**a**) Mg, (**b**) α-Ti, and (**c**) α-Zr alloy. The region bounded by the white line gains charge, while the region bounded by the black line loses charge. The arrow represents the direction of shear on each slip system.

A comparison of the stress–strain curves of Mg and α-Ti/Zr reveals that the addition of hydrogen leads to a reduction in the shear modulus of these materials across all slip directions. However, it should be noted that the effect of hydrogen on the ISS of Mg differs from that observed in α-Ti/Zr. Specifically, while hydrogen reduces the ISS of α-Ti/Zr across all slip systems, the effect of hydrogen on the ISS of Mg varies with the slip systems involved. To gain insight into the impact of hydrogen on the ISS, the transfer of charge after the addition of hydrogen was investigated. Figure 5 presents the charge density difference on the basal, prismatic, pyramidal I, and pyramidal II slip systems around the hydrogen atom at the corresponding maximum shear stress in the Mg, α-Ti and α-Zr alloy. The region bounded by the white line gains charge, while the region bounded by the black line loses charge. Figure 5a depicts the redistribution of charge after the addition of hydrogen in the Mg alloy. The addition of hydrogen leads to a loss of charge between the Mg atoms along the direction of shear on the basal and pyramidal II slip systems, which reduces the bond strength of Mg-Mg, thereby leading to a decrease in the ISS. Conversely, the charge accumulation in the region between the Mg atoms in the direction of shear across the prismatic and pyramidal I slip systems enhances the bond strength of Mg-Mg, leading to an increase in the ISS. Figure 5b illustrates the redistribution of charge after the addition of hydrogen in the Ti alloy. The transfer of charge from the Ti atoms to the hydrogen atom causes a reduction in the bond strength of Ti-Ti in each slip direction, resulting in a decrease in the ISS. Similarly, as shown in Figure 5c, the transfer of charge from the Zr atoms to the hydrogen atom in the Zr alloy results in a decrease in the bond strength of Zr-Zr in each slip direction, leading to a reduction in the ISS.

**Figure 6:**
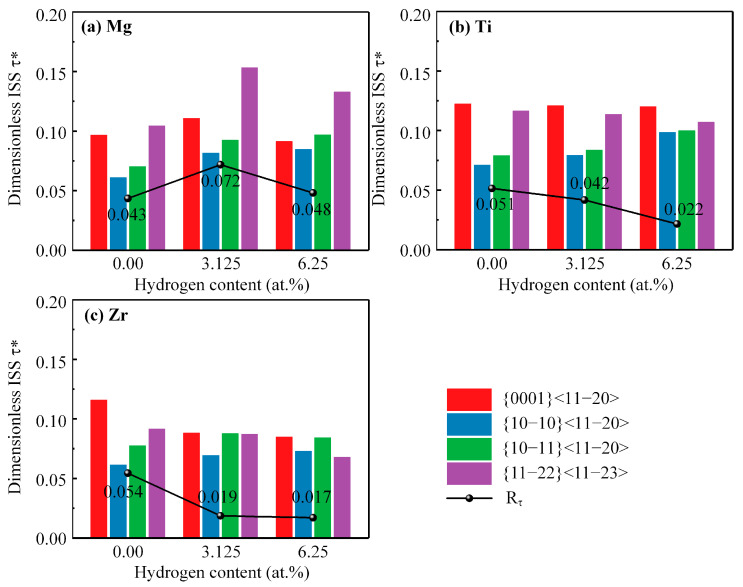
The dimensionless ideal shear strength $\tau^{*}$, along different slip systems and activation anisotropy index $R_{\tau }$ of Mg and α-Ti/Zr. (**a**) Mg, (**b**) α-Ti, and (**c**) α-Zr.

Both shear modulus *G* and ISS are crucial for predicting the activation of slip systems, and the effect of hydrogen on the shear modulus and ISS is different in this work. Therefore, relying on either the shear modulus or ISS alone is insufficient to assess the effect of hydrogen on the slip system activation. Dimensionless ideal shear strength $\tau^{*}$, which combines the ISS and shear modulus, is a normalized measure of the resistance to activate a particular slip system. It is defined as the ratio of the ideal shear strength τ to the shear modulus *G* along a particular slip system. This dimensionless quantity is useful because it allows for direct comparison of the relative ease of slip between different slip systems within the same material. A lower value of dimensionless ISS indicates an easier slip, while a higher value indicates a greater resistance to the slip. As depicted in Figure 6a, the introduction of hydrogen into Mg increases the dimensionless ISS along all slip systems considered, indicating that hydrogen hinders the activation of these slip systems compared to pure Mg. Nevertheless, the hierarchy of the resistance to activate these slip systems remains unaltered. Additionally, the activation anisotropy can be quantified by the index $R_{\tau }=\max\left\{\tau_{i}^{*}\right\}-\min\left\{\tau_{i}^{*}\right\}$, where $\max\left\{\tau_{i}^{*}\right\}$ and $\min\left\{\tau_{i}^{*}\right\}$ represent the maximum and minimum values of the dimensionless ISS $\tau_{i}^{*}$ among the considered slip systems, respectively. The presence of hydrogen in Mg leads to an elevation in the $R_{\tau }$ index, implying that hydrogen enhances the activation anisotropy of these slip systems in a Mg alloy. As illustrated in Figure 6b, an increase in the hydrogen content in the α-Ti alloy results in a gradual increase in the dimensionless ISS across the prismatic and pyramidal I slip systems, whereas a decreasing trend is observed in the dimensionless ISS across the basal and pyramidal II slip systems. This indicates that hydrogen hinders the activation of prismatic and pyramidal I slip systems, while promoting the activation of basal and pyramidal II slip systems. As a result, the index $R_{\tau }$ decreases with an increasing hydrogen content, indicating that hydrogen weakens the activation anisotropy of the slip system in the α-Ti alloy. As shown in Figure 6c, the effect of hydrogen in α-Zr is comparable to that in α-Ti, where the dimensionless ISS across the prismatic and pyramidal I slip systems increases, while the dimensionless ISS across the basal and pyramidal II slip systems decreases as the hydrogen content increases. As a result, hydrogen reduces the index $R_{\tau }$, suggesting that hydrogen reduces the activation anisotropy of the slip systems in the α-Zr alloy. Hence, the reduction in the activation anisotropy would activate multiple slip systems during the plastic deformation, thus enhancing the ductility of α-Ti/Zr.

**Figure 7:**
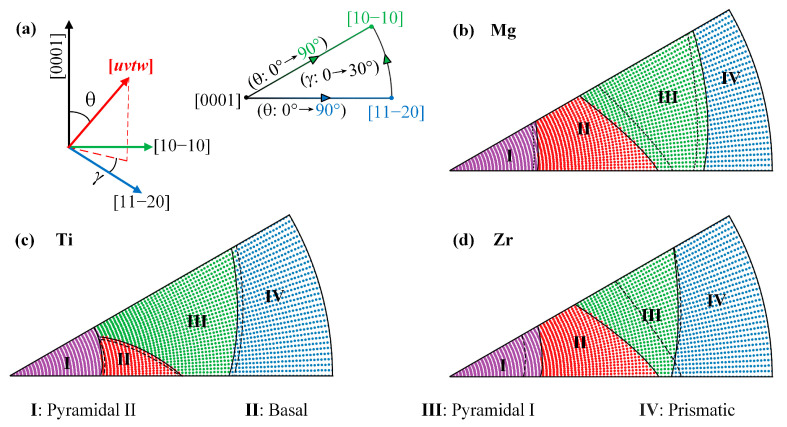
The evolution of the regions corresponding to each slip system before (solid lines) and after (dotted lines) the introduction of hydrogen. (**a**) Schematic diagram of the various tensile loading directions, (**b**) Mg, (**c**) α-Ti, and (**d**) α-Zr.

The activation of the slip system is also dependent upon the relative angle between the loading direction and grain orientation when the grain is under uniaxial tension. Therefore, the activation of slip systems in Mg and α-Ti/Zr alloys subjected to uniaxial tension is discussed in this section. The ISS and Schmidt’s law were utilized to evaluate the minimum tensile stress required to activate the slip system when the grain is subjected to tensile stresses in various directions. The minimum tensile stress $\sigma^{\left[uvtw\right]}$ in a given loading direction (*uvtw*) is calculated as follows: $\sigma_{i}^{\left[uvtw\right]}=\tau_{i}^{CRSS}/m_{i}^{\left[uvtw\right]}$, where $\tau_{i}^{CRSS}$ is the critical resolved shear stress, corresponding to the ISS across a given slip system in a defect-free crystal, and $m_{i}^{\left[uvtw\right]}$ is the Schmid factor for the given slip system when the loading direction is (*uvtw*). The calculation details of the Schmid factors were previously described in the work of Wang [33]. The tensile loading directions considered were all mapped onto a stereographic triangle, as shown in Figure 7, with the corners of the triangle representing the loading directions for [10-10], [11-20], and [0001]. The black solid (without hydrogen) or dotted (with hydrogen) contour lines divide the triangle into four regions. The region I, II, III, and IV correspond to the proportion of the loading directions where pyramidal II, basal, pyramidal I, and prismatic slip systems are the most active slip system requiring the lowest tensile stress, respectively. The proportion of the loading directions represents the activation probability $p_{i}$ of the individual slip systems in polycrystalline materials subjected to uniaxial tension with randomly distributed grain orientation. The plastic anisotropy of polycrystalline materials with randomly distributed grain orientations can be quantified by the index $R_{\sigma }=\max\left\{p_{i}\right\}-\min\left\{p_{i}\right\}$, where $\max\left\{p_{i}\right\}$ and $\min\left\{p_{i}\right\}$ denote the maximum and minimum activation possibility $p_{i}$ of the four slip systems, respectively. Figure 7b illustrates the impact of hydrogen on the proportion of regions I–IV of Mg. The introduction of hydrogen leads to an increase in the proportion of regions II and IV, while simultaneously reducing the proportion of regions I and III. These observations suggest that the presence of hydrogen promotes the activation possibility of the basal and prismatic slip system, while simultaneously reducing the activation possibility of the pyramidal I and pyramidal II slip in polycrystalline Mg with grains that are randomly orientated during the uniaxial tension. However, hydrogen increases the index $R_{\sigma }$ from 11.0% to 12.6%, implying that hydrogen leads to an increase in the plastic anisotropy of polycrystalline Mg subjected to uniaxial tension. Figure 7c demonstrates the evolution of the regions corresponding to each slip system before and after the introduction of hydrogen in α-Ti. Specifically, hydrogen increases the proportion of regions I and III, while simultaneously decreasing the proportion of regions II and IV. Therefore, hydrogen increases the activation possibility of the pyramidal II and pyramidal I slip systems and decreases the activation possibility of the basal and prismatic slip systems. Interestingly, the presence of hydrogen results in a reduction in the index $R_{\sigma }$ from 10.0% to 7.9%. This result suggests that hydrogen would reduce the plastic anisotropy of α-Ti alloy and give rise to more homogeneous plastic flow when the polycrystalline α-Ti is subjected to uniaxial tension. Similar to the role of hydrogen in Mg, the presence of hydrogen in α-Zr increases the proportion of region II but decreases the proportion of regions I and III, as demonstrated in Figure 7d. Therefore, hydrogen increases the activation possibility of the basal slip system and decreases the activation possibility of the pyramidal II and pyramidal I slip systems. Additionally, the presence of hydrogen increases the index $R_{\sigma }$ from 9.8% to 30.3%, indicating hydrogen increases the plastic anisotropy of α-Zr alloy. Therefore, basal slip dominates the plastic deformation in polycrystalline Mg and α-Zr, leading to inhomogeneous plastic deformation and texture.

## 4. Conclusions

The present study employed first-principles calculations to investigate the influence of hydrogen on the ideal shear strength of various slip systems in Mg and α-Ti/Zr. The results indicate that the presence of hydrogen leads to a reduction in the ideal shear strength of Mg across basal and pyramidal II slip systems, as well as α-Ti/Zr across all four systems, but increases the ideal shear strength of Mg across prismatic and pyramidal I slip systems. The dimensionless ideal shear strength was utilized to evaluate the activation anisotropy of these slip systems, revealing that hydrogen increases the activation anisotropy in Mg, while decreasing it in α-Ti/Zr. Furthermore, the effect of hydrogen on the plastic anisotropy of polycrystalline Mg/α-Zr and α-Ti alloys under uniaxial tension was studied, and it was observed that hydrogen enhances the plastic anisotropy of the Mg/α-Zr alloy but reduces it in the α-Ti alloy.

## Supplementary Materials

The following supporting information can be downloaded at: https://www.mdpi.com/article/10.3390/ma16083016/s1, Table S1: The calculated lattice parameters and the solution energy of interstitial hydrogen at the tetrahedral interstitial site (T) and octahedral interstitial site (O) in Mg and α-Ti/Zr.
