# Supplementary material for: The Effect of Hydrogen on Plastic Anisotropy of Mg and α-Ti/Zr from First-Principles Calculations
Source: Materials (Basel). 2023 Apr 11;16(8):3016. doi: 10.3390/ma16083016 (PMC10143018; doi:10.3390/ma16083016)
Supplement: Supplementary file 1 [file materials-16-03016-s001.zip › materials-2312008-supplementary.pdf]

Supplementary

Table S1 The calculated lattice parameters and the solution energy of interstitial hydrogen at the tetrahedral interstitial site (T) and octahedral interstitial site (O) in Mg and α-Ti/Zr.

	Hydrogen content (at. %)	a (Å)	c/a	E_T (eV)	E_O (eV)
Mg	0	3.189	1.629	-	-
	3.125	3.185	1.632	0.289	0.415
	6.25	3.193	1.634	0.178	0.311
α-Ti	0	2.936	1.583	-	-
	3.125	2.939	1.586	-0.351	-0.446
	6.25	2.946	1.587	-0.348	-0.467
α-Zr	0	3.230	1.599	-	-
	3.125	3.234	1.602	-0.459	-0.405
	6.25	3.235	1.608	-0.457	-0.427
